# Induced cardiac pacemaker cells survive metabolic stress owing to their low metabolic demand

**DOI:** 10.1038/s12276-019-0303-6

**Published:** 2019-09-13

**Authors:** Jin-mo Gu, Sandra I. Grijalva, Natasha Fernandez, Elizabeth Kim, D. Brian Foster, Hee Cheol Cho

**Affiliations:** 1 Department of Pediatrics, Emory University, Atlanta, GA 30322 USA; 2 Department of Biomedical Engineering, Georgia Institute of Technology and Emory University, Atlanta, GA 30322 Georgia; 3 Cedars-Sinai Medical Center, Los Angeles, CA 90048 USA; 4 Division of Cardiology, Department of Medicine, The Johns Hopkins University School of Medicine, Baltimore, MD 21205 USA

**Keywords:** Genetic transduction, Cardiovascular biology

## Abstract

Cardiac pacemaker cells of the sinoatrial node initiate each and every heartbeat. Compared with our understanding of the constituents of their electrical excitation, little is known about the metabolic underpinnings that drive the automaticity of pacemaker myocytes. This lack is largely owing to the scarcity of native cardiac pacemaker myocytes. Here, we take advantage of induced pacemaker myocytes generated by TBX18-mediated reprogramming (TBX18-iPMs) to investigate comparative differences in the metabolic program between pacemaker myocytes and working cardiomyocytes. TBX18-iPMs were more resistant to metabolic stresses, exhibiting higher cell viability upon oxidative stress. TBX18-induced pacemaker myocytes (iPMs) expensed a lower degree of oxidative phosphorylation and displayed a smaller capacity for glycolysis compared with control ventricular myocytes. Furthermore, the mitochondria were smaller in TBX18-iPMs than in the control. We reasoned that a shift in the balance between mitochondrial fusion and fission was responsible for the smaller mitochondria observed in TBX18-iPMs. We identified a mitochondrial inner membrane fusion protein, Opa1, as one of the key mediators of this process and demonstrated that the suppression of Opa1 expression increases the rate of synchronous automaticity in TBX18-iPMs. Taken together, our data demonstrate that TBX18-iPMs exhibit a low metabolic demand that matches their mitochondrial morphology and ability to withstand metabolic insult.

## Heart health: understanding pacemaker cells

The heart’s pacemaker cells contain mitochondria that are smaller than average and require less energy than other heart cells, properties that help make them naturally resilient to stress. Cardiac pacemaker cells constitute a tiny proportion of the heart’s cells, yet play a critical role in maintaining a steady heartbeat. However, quite how pacemaker cells maintain their automatic rhythm is unclear because their scarcity makes them difficult to study. To examine the cells’ metabolic state further, Hee Cheol Cho at Emory University, Atlanta, and Brian Foster at Johns Hopkins University School of Medicine, Baltimore, and co-workers therefore induced pacemaker cells by adding an embryonic protein to heart muscle cells. The induced pacemaker cells survived well under oxidative stress. The team identified a protein in the pacemakers’ mitochondrial membranes, the expression of which directly influences rhythm responses.

## Introduction

Cardiac pacemaker cells in the sinoatrial node (SA node or SAN) initiate every heartbeat with spontaneous membrane depolarization orchestrated by the sarcolemmal ion channels^1^ and rhythmic intracellular Ca^2+^ release events from the sarcoplasmic reticulum^2^. Embryonic development of the SAN is distinct from that of the chamber myocardium, where T-box transcription factors, TBX3, TBX5, TBX18, and a homeodomain transcription factor, Shox2, guide the formation of the compact pacemaker node structure^3,4^. The compact node shows an unusually high content of nonmyocytes compared with the working myocardium^5^. Within the SA node, action potential propagation is unusually slow owing to the expression of a low conductance gap junction, Cx45, at the expense of Cx43, which is prevalent throughout the chamber myocardium^6^. The pacemaker cells are uniquely shaped, with elongated morphology and disorganized sarcomere structures^4^, suggesting that the contractility of the pacemaker cells may be weaker than that of the chamber cardiomyocytes.

The myocardium is one of the most energy-demanding organs; an adult human heart utilizes ~ 6 kg of total recycled ATPs each day^7–9^. As the chamber cardiomyocytes become mature, the main ATP-generating metabolism switches from oxidative glycolysis to fatty-acid oxidation^10^. Under a low oxygen supply, anaerobic glycolysis provides ATP and maintains cardiomyocyte viability but generates lactate as a byproduct. Accumulation of excess lactate contributes to acidic pH and mitochondrial damage, which are the main causes of myocardial cell death upon hypoxic stress^11–13^.

Mitochondria generate > 90% of total ATP production via oxidative phosphorylation (OxPhos) machinery sequestered in the mitochondrial inner membrane^14^. Mitochondrial membrane dynamics are critical to maintaining the function of these organelles and undergo constant fusion and fission, regulated by Dynamin-related GTPases^15^. In cardiac myocytes, outer mitochondrial membrane (OMM) fusion is regulated by mitofusins 1 and 2 (Mfn1 and Mfn2)^16^. Mfn1 and Mfn2 share > 70% sequence identity with two heptad repeats (HR1 and HR2), sequence motifs forming a helical coiled-coil structure, and a GTPase domain^17^. The HR2 domain at the Mfn C-terminus dimerizes with another HR2 to tether the adjacent mitochondrial outer membranes^18^. The GTPase-dependent conformational changes of Mfns are required to complete the OMM fusion process^19^. Inner mitochondrial membrane (IMM) fusion is regulated by optic atropy (Opa1)^20^, which contributes to cristae morphogenesis and OxPhos efficacy^21,22^. Global knockdown of long and short forms of Opa1, generated by OMA1 and Yme1l1 protease activity^23^, leads to fragmented mitochondria^20,24^. Fusogenic events of IMM also require the GTPase activity of Opa1^25^. Conversely, fission is orchestrated by dynamin-related protein 1 (Drp1) and adaptor proteins, such as mitochondrial fission factors (Mff) and mitochondrial fission protein (Fis1)^26^. Accumulating evidence suggests that mitochondrial dynamics per se can remodel cellular metabolism^27^.

Previous studies have observed that the isolated, native SA node exhibited remarkable resilience to prolonged hypoxia followed by reoxygenation^28–30^, a condition that would irreversibly damage chamber cardiomyocytes. However, the minuscule size of the native SAN and the scarcity of the native pacemaker cells within it have impeded progress in the understanding of this phenomenon. We have previously demonstrated that somatic gene transfer of an embryonic transcription factor, TBX18, suffices to reprogram ordinary ventricular myocytes (VMs) to induced pacemaker myocytes (iPMs) in vitro and in vivo, recapitulating the hallmark features of native pacemaker cells^31–36^. We asked whether the iPMs display resistance to hypoxic stress similar to the native pacemaker tissue and set out to characterize the general metabolic properties of TBX18-iPMs. We examined oxygen consumption rates (OCRs) and glycolysis substrate/byproduct contents in TBX18-iPMs and analyzed the morphological features of mitochondria in iPMs compared with control ventricular myocytes. We draw parallels between the metabolic features of the iPMs and those of the native pacemaker cells and present data showing that the automaticity of iPMs is influenced by the degree of mitochondrial fusion/fission.

## Materials and methods

### Animal care and neonatal rat ventricular myocyte (NRVM) isolation

Pregnant Sprague Dawley rats were purchased from Charles River Laboratories (Wilmington, MA) and maintained in accordance with the Emory University Institutional Animal Care and Use Committee (IACUC). NRVMs were isolated from 1- to 3-day-old pups and cultured as a monolayer as described previously^31^. Only the apical half of the neonatal ventricle was excised for cell culture to minimize contaminating atrioventricular nodal cells. NRVMs were transduced with either Ad-TBX18-IRES-ZsGreen1 or Ad-GFP at multiplicity of infection of one fluorescence-forming unit (ffu) per cell^37^. Routine NRVM culture media, based on M199 supplemented with 10 mm HEPES, 0.1 mm nonessential amino acids, 3.5 mg/mL glucose, 2 mm l-glutamine, 4 μg/ml vitamin B_12_, 100 U/ml penicillin and heat-inactivated fetal bovine serum at 10% (first 2 days of culture) or 2% (after 2 days of culture) final concentration, was used.

### OCR measurement

The OCR was measured with a SeaHorse XFp extracellular flux analyzer (SeaHorse Bioscience). All assays were performed according to the manufacturer’s protocol (SeaHorse Bioscience). In brief, GFP- and TBX18-NRVMs were plated onto XFp cell culture miniplates. One day before the assay, XFp sensor cartridges were calibrated with calibration buffer overnight at 37 °C in an incubator without CO_2_. On the assay day, the media was replaced with SeaHorse assay media, and the plate was incubated at 37 °C in an incubator without CO_2_ for 45 min immediately before the assay to allow medium temperature and pH to reach equilibrium. The initial titration of carbonyl cyanide p-(trifluoromethoxy) phenylhydrazone (FCCP) was performed with 2 µm FCCP for all assays.

### Lactate and glycogen measurement

Lactate and glycogen contents were measured with commercially available kits (Sigma-Aldrich, Cat# MAK064 and Cat# MAK016, respectively) according to the manufacturers’ instructions. For glycogen storage quantification, the cells were plated on 24-well plates and harvested at predetermined time points. The cells were lysed in boiling water for 5 min and centrifuged. The supernatant was transferred to a 96-well plate. The glycogen content was estimated indirectly by measuring the hydrogen peroxide content, which is generated from the serial reaction of glycogen in the supernatant with amyloglucosidase and glucose oxidase provided in the kit, which in turn drives horseradish peroxidase (HRP) activity in a colorimetric assay at 570 nm.

For lactate content measurements, the media was replaced with phenol-free 2% serum NRVM media 1 day before collecting the media. Media containing secreted lactate were collected and filtered by spin columns with a 10 kDa cutoff. The lactate content was estimated indirectly by measuring the hydrogen peroxide content generated from the oxidation of lactate in the media with lactate oxidase provided in the kit, which in turn drives HRP activity in a colorimetric assay at 570 nm. A standard curve was generated for each assay with known concentrations of glycogen or lactate.

### Ethidium D homodimer-1 (EthD-1) assay

NRVMs were transduced with Ad-GFP or Ad-TBX18-IRES-ZsGreen1 and plated onto 96-well plates. For all hypoxia studies, we cultured the myocytes in a dual-gas hypoxia incubator, in which we increased the N_2_ content to reduce the O_2_ content to 1%, and the normoxia control myocytes were cultured in 20% O_2._ The CO_2_ content was maintained at 5% for all experiments to maintain the pH of the bicarbonate-based buffer in the media. The media were replenished daily from D3 to D6, during which metabolic stresses were imposed. On D6, the cells were incubated with EthD-1 and Hoechst 33342 for 20 min in a 37 °C 5% CO_2_ incubator. The percentage of cell death was calculated by counting the number of nuclei positive for EthD-1 normalized to the total number of nuclei positive for Hoechst 33342. Images were taken from one field per well and *n* ≥ 8 replicate wells per condition and were analyzed with CellProfiler.

### siScramble and siOpa1 transfection

Scramble-siRNA (Ambion, Cat# AM4611) or On-Target plus rat siRNA against Opa1 (Dharmacon, Cat# L-086996-02-0005; 5′-GGAAGAUCUUGCAGCGUUA-3′, 5′-GCAUACAUUUGAAGAACGA-3′, 5′-GUUCAGAAGACCUCGCCAA-3′, 5′-GCUGAUAGUUUUAAAGCGA-3′) at 15 or 30 pmol were transfected into NRVM monolayers with Viromer Blue (Origene) 1 day after transducing the myocytes with either Ad-GFP or Ad-TBX18-IRES-ZsGreen1. Media containing the transfection reagents were replaced with fresh media 6 h after transfection.

### mRNA extraction and real-time PCR

The mRNA of Ad-GFP or Ad-TBX18-IRES-ZsGreen1-transduced NRVMs were extracted with Qiazol (Qiagen, Hilden, Germany) according to the manufacturer’s instructions. The mRNA samples were converted to first strand cDNA using a Takara reverse transcription kit (Takara Bio Inc., Kusatsu, Japan). Then, real-time PCR was performed by using a 2X SYBR mix (Qiagen) with Rotor-Gene Q (Qiagen, Hilden, Germany). The relative expression of the genes was calculated by normalizing with the housekeeping genes. The primer sequences and catalog numbers are as follows: *Gapdh* Forward, 5′-CCCATCACCATCTTCCAGG-3′; *Gapdh* Reverse, 5′-GAGCCCCAGCCTTCTCCATG-3′; *Oma1* Forward, 5′-TCAAGATGTCCCAGGGGTCT-3′; *Oma1* Reverse, 5′-CTTGTCCGTTTGGAAGCACG-3′; *Yme1l1* Forward, 5′-GACACGGCGTTGCAATCTA-3′; *Yme1l1* Reverse, 5′-CCCGCAAAAGAAACCCCTTC-3′; *Opa1* (Thermo Fisher, Cat# QT00177597); *Mfn1* (Thermo Fisher, Cat# QT01080898); and *Mfn2* (Thermo Fisher, Cat# QT00183337).

### Western blot analysis

All protein samples were prepared with radioimmunoprecipitation assay buffer (Thermo Scientific) containing a protease and phosphatase inhibitor mixture (Thermo Scientific). Protein content was quantified by BCA assay, and the cell lysates were run on a 10% or 12% sodium dodecyl sulfate-polyacrylamide gel and transferred onto a polyvinylidene difluoride (PVDF) membrane. Then, the transferred membrane was incubated with a primary antibody overnight at 4 °C, followed by a 1 h incubation with a green or far-red fluorophore-conjugated secondary antibody (Li-Cor Biosciences, Lincoln, NE). Western blots were performed using specific antibodies against TBX18 (Santa Cruz Biotechnology, Inc., Dallas, TX), Opa1 (BD Biosciences, San Jose, CA), Cx43 (Sigma-Aldrich, St. Louise, MO), p-Cx43 (Cell Signaling Technologies, Danvers, MA), and Cx45 (rabbit sera kindly donated by Dr. Koval). The Odyssey CLx imaging system (Li-Cor Biosciences, Lincoln, NE) was used to detect immunoreactivity. The PVDF western blot membranes were stripped and reblotted with a monoclonal anti-GAPDH antibody (Bio-Rad Laboratories, Hercules, CA) for loading control and normalization.

### Multielectrode analysis (MEA) array and analysis

NRVMs expressing either TBX18 or GFP were plated on 48-well MEA plates and transfected with either siScramble or siOpa1 RNA the following day. Extracellular field potential signals from a monolayer of NRVMs were measured with the Maestro System (Axion Biosystems, Atlanta, GA) at the given time points. The conduction velocity and beats per minute were obtained from the synchronous beats that have activity on more than half of the total electrodes.

### Mitochondrial size analysis

To quantify the mitochondrial size, GFP-NRVMs and TBX18-iPMs were stained with 25 nm MitoTracker Red CMXRos (Cat# M7512, Molecular Probes, Eugene, OR) according to the manufacturer’s instructions. On D3 after adenoviral transduction or 48 h. after siRNA transfection, the cells were washed with PBS and stained with MitoTracker Red CMXRos for 20 min. The DeltaVision OMX Super Resolution Imaging System (GE Healthcare Life Sciences, Marlborough, MA) was used to acquire superresolution images of mitochondria. For mitochondrial imaging of native cells, mouse ventricular myocytes and pacemaker cells were freshly isolated as previously described^38,39^. Image analysis was performed with ImageJ software, and 3-D image visualization and analysis were performed with Imaris software (Bitplane, Belfast, UK).

### Mass spectrometry analysis

Protein samples collected from GFP-NRVMs and TBX18-iPMs were precipitated, reduced, alkylated, and digested as previously described^40^. Peptides were desalted using tC18 Sep Pak cartridges (Waters) and evaporated to dryness before resolubilizing and labeling with a tandem mass tagging reagent (TMT, Thermo Fisher Scientific, Waltham, MA) according to the manufacturer’s instructions. Peptides were subjected to high-pH reversed-phase high-pressure liquid chromatography (bRP-HPLC) prior to low pH RP-HPLC coupled to tandem mass spectrometry (LC-MS/MS) on a Q-Exactive Hybrid Quadrupole-Orbitrap Mass Spectrometer (Thermo Fisher). Peptides and proteins were identified by searching a rat Refseq database (29449 sequences) using Mascot 2.2.0 (Matrix Science) interfaced through Proteome Discoverer 1.4 (Thermo Fisher Scientific). TMT signals were quantified using a median sweep algorithm as previously described^40–42^. An empirical Bayes-modified two-sample *t* test was used for statistical analysis^40,42,43^.

### Quantification and statistical analysis

Two-tailed Student’s *t* tests were used to calculate the statistical significance, and *p* < 0.05 was considered to be significant. All error bars represent the mean ± the standard error of the mean.

## Results

### Induced pacemaker cells are viable upon chronic metabolic stress

**Fig. 1:**
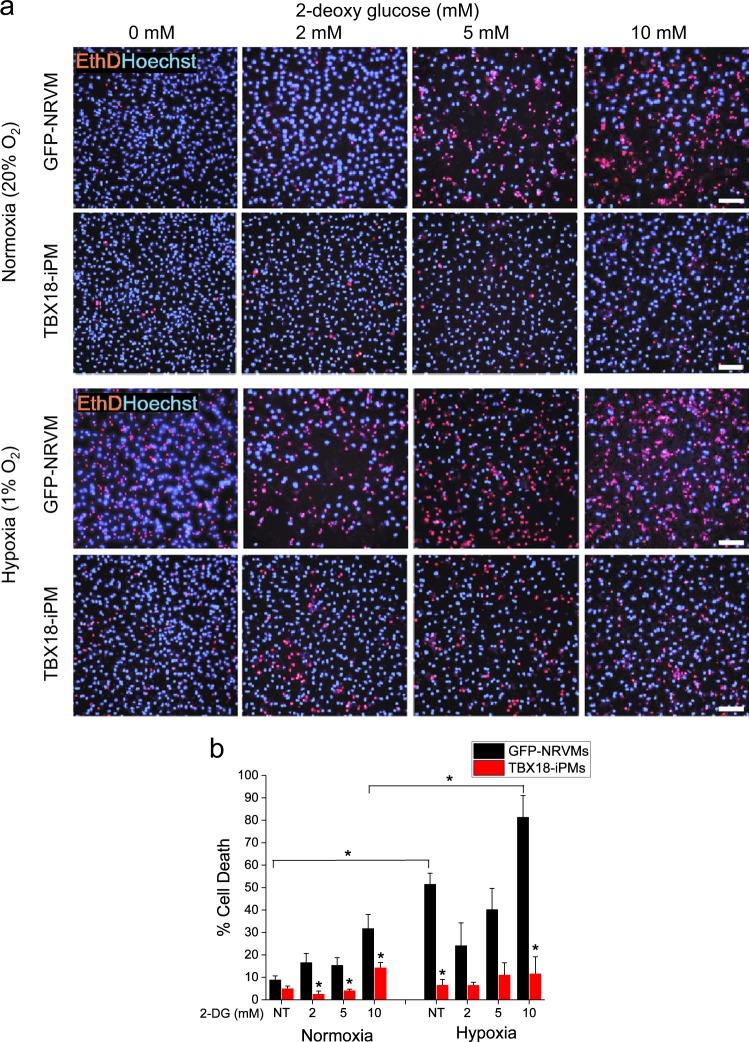
Higher resistance of TBX18-iPMs to metabolic stresses. **a** TBX18- and GFP-NRVMs were incubated in normoxia or 1% oxygen (Hypoxia) with or without 2-DG treatment for 3 days from D3 to D6, followed by ethidium homodimer-1 (EthD-1) staining on D6. The % cell death was calculated based on the number of EthD-1^+^ cells per total cells. Representative pictures of EthD-1 and Hoechst 33342 staining of D6 GFP- and TBX18-NRVMs. **b** Quantitative graphs of % cell death (*n* ≥ 8 for each condition). Scale bar: 10 µm *p < 0.05

We hypothesized that TBX18-iPMs survive better than control, Ad-GFP-transduced neonatal rat ventricular myocytes (GFP-NRVMs) do under metabolic stresses. To test this hypothesis, GFP-NRVMs and TBX18-iPMs were cultured for 3 days under normoxia, followed by an additional three days of culture under hypoxia at 1% O_2_. On day 6 (D6), the myocytes were stained with ethidium homodimer-1 (EthD-1), a cell-impermeant viability indicator that crosses damaged membranes and emits red fluorescence upon binding to DNA. This chronic hypoxic stress led to >50% cell death in control GFP-NRVMs, as anticipated from the dependence of ventricular myocytes on oxidative phosphorylation. Strikingly, the viability of the iPMs was largely unaffected by chronic hypoxia, maintaining > 90% viability after 3 days of severe hypoxic stress (*n* = 8) (Fig. 1a left panels, Fig. 1b nontreated (NT) bars). We asked whether the inhibition of glucose metabolism in the absence or presence of chronic hypoxia would lead to the cell death of the iPMs by treating the myocytes from D3 to D6 with 2-deoxy-d-glucose (2-DG), a glucose antagonist that competitively inhibits the production of glucose-6-phosphate from glucose. As expected, control NRVMs showed increased cell death with increasing doses of 2-DG under normoxia (*n* = 10) (Fig. 1a, top panel). In contrast, no significant increase in cell death was observed in TBX18-iPMs up to 5 mm 2-DG (*n* = 10) (Fig. 1a second row, Fig. 1b left panel). When glucose metabolism was inhibited under chronic hypoxia, control NRVMs showed a trend of higher cell death at respective 2-DG concentrations (*n* = 8) (Fig. 1a third row). Even under the combined metabolic stresses, TBX18-iPMs were largely viable, exhibiting >85% viability with 10 mm 2-DG and under chronic hypoxia (*n* = 8) (Fig. 1a fourth row, Fig. 1b right panel).

### The mitochondria in induced pacemaker cells are unusually small and globular

**Fig. 2:**
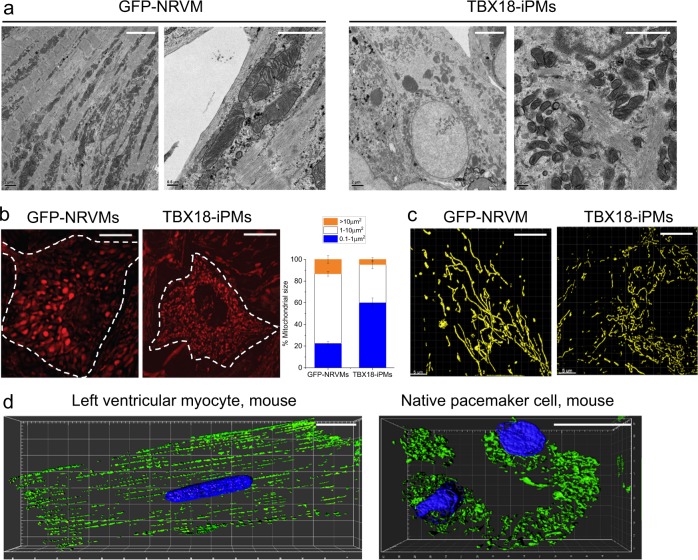
Smaller mitochondria in TBX18-iPMs and native pacemaker cells. **a** Representative EM images of mitochondria with two different magnifications from GFP-NRVMs and TBX18-iPMs. **b** Mitochondria size distribution of GFP-NRVMs and TBX18-iPMs. Representative images of MitoTracker staining (left) and percentage of mitochondria according to their size distribution in three groups: >10 .m^2^, 1–10 µm^2^, and 0.1–1 µm^2^ (right) (*n* = 10 for GFP-NRVMs and *n* = 14 for TBX18-iPMs). **c** Representative superresolution images of mitochondria stained with MitoTracker from GFP-NRVMs and TBX18-iPMs. **d** Representative superresolution images of mitochondria stained with MitoTracker from freshly isolated mouse ventricular myocytes and pacemaker cells. Scale bar: 2 µm (a: right panels of each group), 5 μm (**a**: left panels of each group and **b**), and 10 μm (**c** and **d**) *p < 0.05

Mitochondria constantly fuse and divide^15^. These fusion and fission events are required to properly distribute and eliminate impaired or damaged mitochondria^44,45^. To investigate the ability of iPMs to withstand chronic and extensive metabolic stress, we first examined the shape and size of the mitochondria of control GFP-NRVMS and TBX18-iPMs. Transmission electron microscopy (TEM) images of the myocytes revealed that the mitochondria were unusually small and globular in TBX18-iPMs, unlike the longitudinal networks of the mitochondria juxtaposed between myofibrils in GFP-NRVMs (Fig. 2a). At a higher magnification, the TEM images also revealed that the mitochondrial cristae were sparse in TBX18-iPMs compared to those in GFP-NRVMs (Fig. 2a). To quantify the size of the mitochondria, we labeled the myocytes with MitoTracker Red CMXRos, which accumulates inside active mitochondria, and performed live cell imaging. The majority of mitochondria in TBX18-iPMs ranged from 0.1 to 1.0 µm^2^ in the cross-sectional area, whereas most of the mitochondria in GFP-NRVMs ranged from 1.0 to 10 µm^2^ in size (*n* = 10 for GFP-NRVMs and *n* = 14 for TBX18-iPMs) (Fig. 2b). We examined whether the smaller cross-sectional area of mitochondria in TBX18-iPMs relates to the smaller volume of the organelles in 3D. The three-dimensional, superresolution images of the MitoTracker-stained mitochondria confirm that the mitochondria in TBX18-iPMs were smaller and globular compared with those in control GFP-NRVMs (Fig. 2c). To investigate whether the comparatively small and globular mitochondria in TBX18-iPMs resemble those in native pacemaker cells from the SA node, left ventricular myocytes and SAN pacemaker cells were freshly isolated, stained with MitoTracker and imaged in 3D. Similar to the small and globular mitochondria observed in TBX18-iPMs, the mitochondria in mouse SAN pacemaker cells were disorganized and irregularly shaped compared with those in ventricular myocytes (Fig. 2d). Small, globular mitochondria with sparse cristae would favor the effective elimination of damaged mitochondria at the expense of decreased output in ATP production^46,47^. This finding prompted us to examine the level of metabolic demand in TBX18-iPMs in relation to chamber cardiomyocytes.

### Induced pacemaker cells are metabolically less demanding

**Fig. 3:**
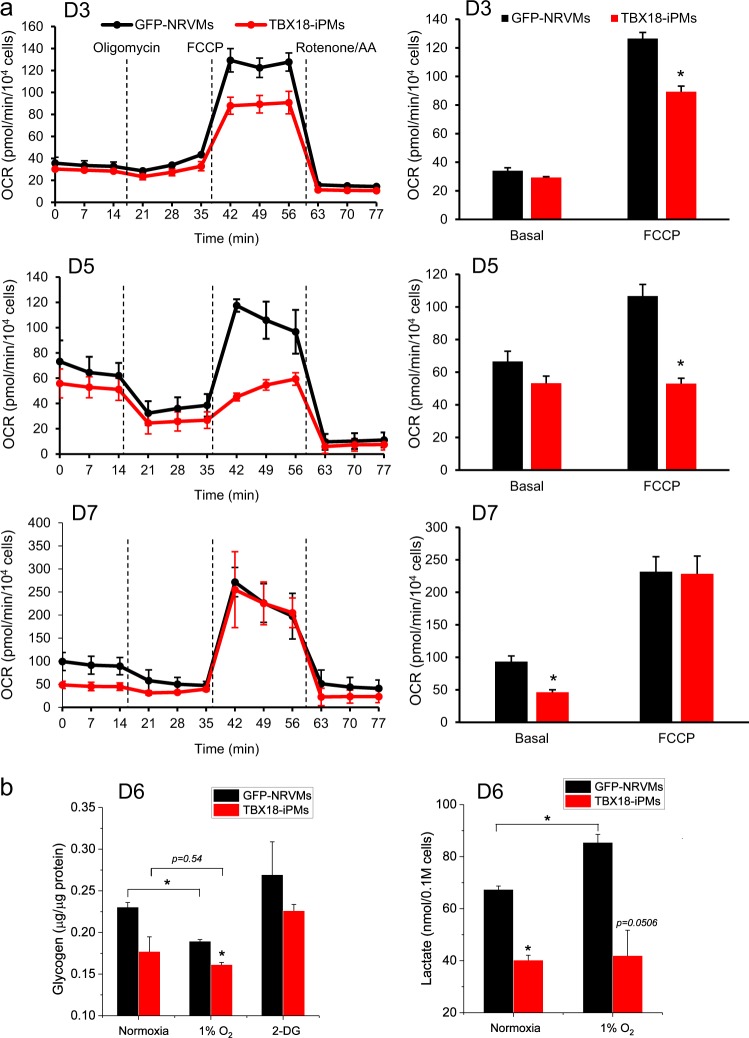
TBX18-iPMs show a slower oxygen consumption rate and a lower glycolytic capacity. **a** Oxygen consumption rate (OCR) of TBX18-iPMs and GFP-NRVMs was measured with a SeaHorse XFp analyzer at baseline and after sequential treatments with oligomycin (an ATP-synthase inhibitor), FCCP (an uncoupler of mitochondrial oxidative phosphorylation), and rotenone/AA (an inhibitor of mitochondrial electron transport chain) on D3, D5, and D7. Basal and maximal OCR are plotted for each time point (*n* = 6). **b** Glycogen contents were determined in the cell lysates of D6 TBX18-iPMs and GFP-NRVMs under normoxia, after 1 hour of incubation in 1% O_2_ or upon treatment with 20 mm 2-deoxy-d-glucose (2-DG) (*n* = 8). The lactate content in the media from D6 TBX18-iPMs and GFP-NRVMs was quantitated under normoxia or after incubating the myocytes in 1% O_2_ for 1 hour (*n* = 8). *p < 0.05

We measured the OCR of GFP-NRVMs and TBX18-iPMs with Seahorse XFp at 3, 5, and 7 days after somatic gene transfer. On D3, the maximal respiratory capacity was significantly lower in TBX18-iPMs compared to control (89 ± 4 vs. 127 ± 4 pmol/min, respectively, *n* = 6, *p* < 0.05), whereas the basal OCR was similar between the two groups (Fig. 3a, top panel). The maximal OCR was still lower in TBX18-iPMs on D5 compared with that in control (53 ± 3 vs. 107 ± 7 pmol/min, respectively, *n* = 6, *p* < 0.05, Fig. 3a, middle panel). On D7, maximal OCR levels were indistinguishable between the two groups, whereas the basal OCR was lower in TBX18-iPMs by ~ 50% compared with the basal OCR in control (46 ± 4 vs. 93 ± 9 pmol/min, respectively, *n* = 6, *p* < 0.05, Fig. 3a bottom panel).

We examined the contents of intracellular glycogen storage and secreted lactate as a measure of glycolysis. TBX18-iPMs showed a trend toward lower levels of glycogen compared with GFP-NRVMs (Fig. 3b, left panel) under normoxia and upon treatment with 2-DG, which would impede glycogen breakdown owing to its accumulation of glucose. Under hypoxia, the cells resort to anaerobic glycolysis to generate ATPs^48^. Upon 1 h of incubation in 1% O_2_, the glycogen content of GFP-NRVMs decreased significantly by 18% under normoxic conditions (*n* = 8). Surprisingly, the glycogen content in TBX18-iPMs under normoxic conditions was not different from that observed under hypoxia for 1 h (*n* = 8, Fig. 3b). This finding suggested that iPMs do not need to engage glycogen breakdown and anaerobic glycolysis during acute hypoxia. We measured the media concentration of lactate, a metabolic byproduct released from the cells that undergo anaerobic glycolysis. Control GFP-NRVMs showed a significantly higher concentration of lactate compared with that of TBX18-iPMs under both normoxic and hypoxic conditions, and the lactate concentration in control myocytes was increased after 1 h of hypoxia, indicating that the ventricular myocytes switched to anaerobic glycolysis upon hypoxia (*n* = 8). In contrast, the lactate concentration in the media of TBX18-iPMs did not increase after 1 h of hypoxia (*n* = 8) (Fig. 3b, right panel). This result is in line with a steady glycogen level in TBX18-iPMs after hypoxia (Fig. 3b, left panel).

### Mitochondrial fission facilitates the synchronous pacing of the induced pacemaker cells

**Fig. 4:**
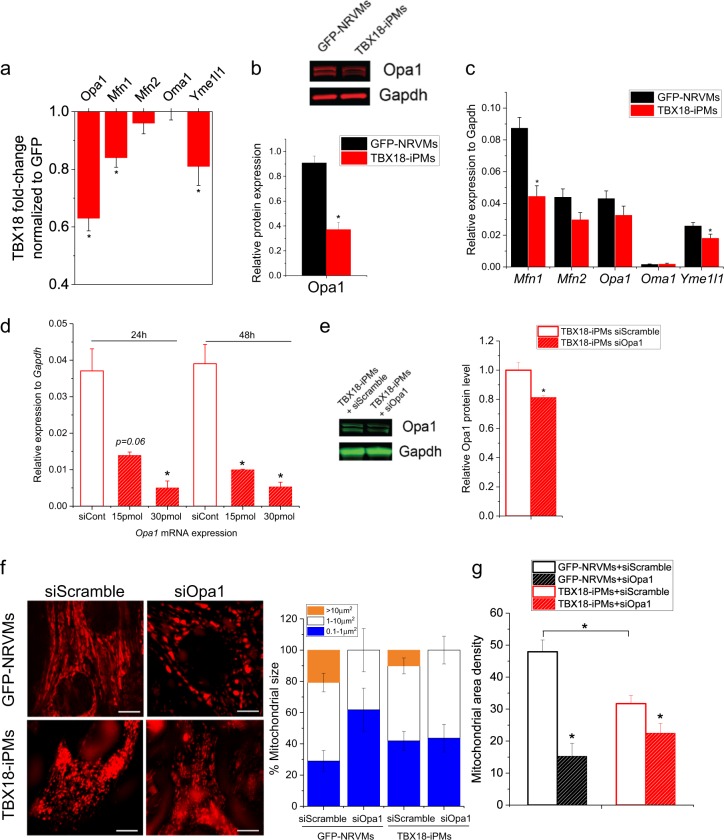
The reduced level of Opa1 in TBX18-iPMs correlates with smaller mitochondria. **a** Proteomic quantification of mitochondrial fusion factors, Mfn1, Mfn2, Opa1, Oma1, and Yme1l1 in the TBX18-iPM proteome (*n* = 3). **b** Validation of Opa1 protein expression levels in D3 GFP-NRVMs and TBX18-iPMs (*n* = 6). **c** Relative mRNA expression levels of fusion-related factors, including *Mfn1*, *Mfn2*, *Opa1*, *Oma1*, and *Yme1l*, normalized to *Gapdh* in GFP-NRVMs and TBX18-iPMs (*n* = 6). **d** mRNA level of *Opa1* after siOpa1 transfection in TBX18-iPMs (*n* = 4). **e** Reduced protein level of Opa1 with siOpa1 transfection in TBX18-iPMs. Representative immunoblot (left) and quantitative graph (right) (*n* = 4). **f** Mitochondrial size distribution of GFP-NRVMs and TBX18-iPMs with siScramble or siOpa1 transfection. Representative pictures of Mitotracker staining (left) and % mitochondrial size distribution divided as >10 μm^2^, 1–10 μm^2^, and 0.1–1 μm^2^ (right) (*n* = 4 for GFP-NRVMs transfected with siScramble or siOpa1 and *n* = 6 for TBX18-iPMs transfected with siScramble or siOpa1). **g** Mitochondrial density in given cytoplasmic area of GFP-NRVMs and TBX18-iPMs with siScramble or siOpa1 transfection (*n* = 4 for GFP-NRVMs transfected with siScramble or siOpa1 and *n* = 6 for TBX18-iPMs transfected with siScramble or siOpa1). *p < 0.05

We asked whether the fragmented mitochondrial morphology and the comparatively low metabolic demand of iPMs are mere consequences of the TBX18-mediated reprogramming of ventricular myocytes to pacemaker cells or whether these features are contributors to the de novo automaticity of iPMs. To gain initial guidance, we performed proteomic analyses of GFP-NRVMs and TBX18-iPMs and examined the differential expression of mitochondrial fusion-related factors, such as Mfn1, Mfn2, Opa1, Oma1, and Yme1l1. Most mitochondrial fusion-related protein levels were downregulated (*n* = 3) (Fig. 4a). From this mass spectrometry analysis, we focused on the most downregulated protein, Opa1, a ubiquitously expressed GTPase that is targeted to the IMM^49^. Quantitative protein measurement by Western blotting confirmed that Opa1 protein levels were downregulated by > 50% in TBX18-iPMs compared with GFP-NRVMs (*n* = 6) (Fig. 4b). Despite the significant reduction in Opa1 at the protein level in TBX18-iPMs, the reduction in *Opa1* at the mRNA level was not statistically significant (*n* = 6) (Fig. 4c).

To determine whether smaller mitochondria contribute to de novo automaticity in TBX18-iPMs, we facilitated mitochondrial fission by knocking down Opa1 protein levels. This experiment was motivated by a previous report that adult heterozygous mice with a missense mutation in Opa1 showed an increase in mitochondrial fission and fragmentation^50^. We tested siRNA constructs against Opa1 (siOpa1) and found that siOpa1 could decrease both transcript and protein levels of Opa1 in TBX18-iPMs in a dose-dependent manner (*n* = 4, Fig. 4d, e). The knockdown of Opa1 with siRNA effectively reduced mitochondrial size and density in both GFP-NRVMs and TBX18-iPMs, in which mitochondria with > 10 µm^2^ in cross-sectional area were no longer visible (*n* = 4 for GFP-NRVMs transfected with siScramble or siOpa1 and *n* = 6 for TBX18-iPMs transfected with siScramble or siOpa1) (Fig. 4f, g).

**Fig. 5:**
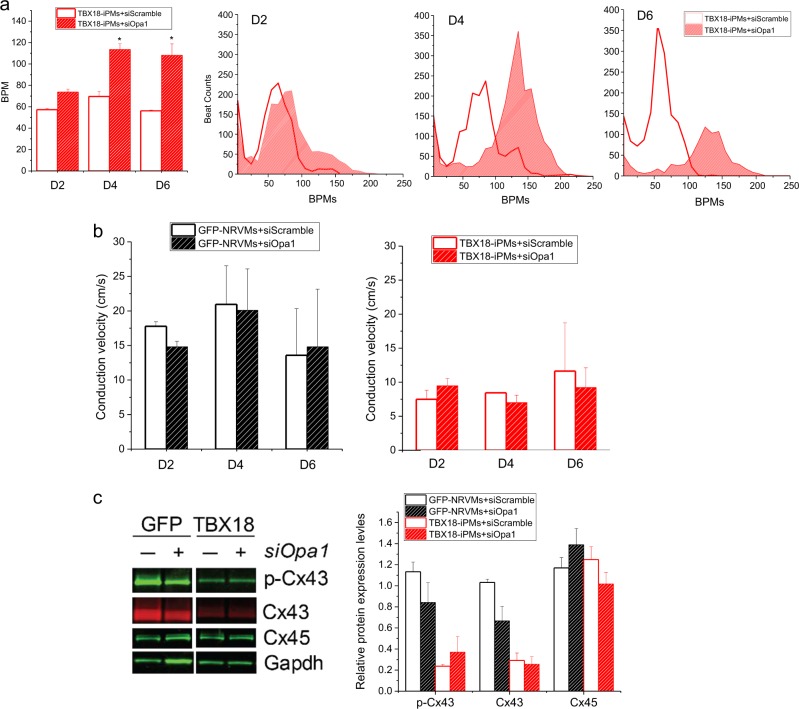
Mitochondrial dynamics regulate the synchronous automaticity of TBX18-iPMs. **a** BPMs of synchronous beats from siScramble- or siOpa1-transfected TBX18-iPMs on D2, D4, and D6 after transfection (*n* = 5). Representative distribution of synchronous beats from siScramble- or siOpa1-transfected TBX18-iPMs on D2, D4, and D6 after transfection (right three panels). **b** Conduction velocity of spontaneous synchronous beatings from GFP- and TBX18-NRVMs with or without Opa1 knockdown on D2, D4, and D6 after transfection (*n* = 5). **c** Relative protein levels of selected gap junctions, including phosphorylated Cx43 (p-Cx43), Cx43, and Cx45, were analyzed in D4 GFP-NRVMs and TBX18-iPMs (*n* = 4). Representative immunoblots are shown on the left and a quantitative graph is presented on the right. BPM: Beats per minute. Scale bar: 5 μm *p < 0.05

We examined pacemaker automaticity by measuring the spontaneous and synchronous electrical activation of TBX18-iPMs by plating iPMs as monolayers on MEAs. For this experiment, we defined spontaneous electrical activities as synchronous pacing when field potentials could be recorded from ≥50% of the 16 total electrodes from each array at 2, 4, and 6 days after siOpa1 transfection. Opa1 knockdown significantly increased the synchronous pacing rate of TBX18-iPMs on D4 and D6 after siOpa1 transfection compared to that of the same cells transfected with control, scrambled siRNA (*n* = 5) (Fig. 5a). Enhanced cell–cell electrical coupling could increase the synchronous pacing observed in TBX18-iPMs under Opa1 knockdown. The measurement of conduction velocities with MEAs indicated that Opa1 knockdown did not influence the conduction velocities of TBX18-iPMs. Similarly, the conduction velocities of GFP-NRVMs did not differ between siOpa1 and siScramble transfection (*n* = 5, Fig. 5b). Gap junction protein levels were unaffected by Opa1 knockdown, including total and phosphorylated connexin 43 (Cx43, p-Cx43) as well as total Cx45, in TBX18-iPMs and in GFP-NRVMs (*n* = 4, Fig. 5c). Taken together, the data suggest that mitochondrial fission facilitates the synchronous pacing of the iPMs without affecting the electrical coupling of the pacemaker cells.

## Discussion

Previous studies have alluded that the sinoatrial node exhibits a remarkable ability to recover from severe hypoxia that would irreversibly damage working myocardium^28,30,51^. The minuscule size of the native cardiac pacemaker tissue has limited biochemical assays aimed at a finer understanding of this initial observation. Here, we took advantage of our induced pacemaker myocyte platform to test whether the ability of the SA node to continuously function through metabolic stress could be recapitulated in TBX18-induced cardiac pacemaker myocytes. Our data demonstrate that TBX18-iPMs are remarkably resilient to chronic and severe metabolic stress, exhibiting negligible cell death after 3 days of near anoxia combined with the inhibition of glycolysis. Similar to the disconnected mitochondria observed in SAN pacemaker myocytes^52^ (Fig. 2d), the mitochondria were smaller and globular in TBX18-iPMs compared with the highly networked interfibrillar mitochondria in the control ventricular myocytes (Fig. 2a–c). The basal OCR, glycogen content, and secreted lactate concentration of iPMs were lower compared with those of ventricular myocytes (Fig. 3), indicating that the global metabolic demand is significantly lower in the pacemaker myocytes.

Mitochondria undergo dynamic fusion and fission events in response to changing energy demand and supply^53^. Mitochondrial fission facilitates the removal of impaired or damaged mitochondria, preventing the spread of mitochondrial membrane depolarization^46,47,54^. Thus, the striking viability of TBX18-iPMs under severe metabolic insults is in line with the small and disconnected mitochondria in the iPMs. The higher degree mitochondrial networks and dense cristae were superior to the globular and disconnected mitochondria in generating maximal ATP per unit area^55^. However, a typical nodal cell appears “empty” owing to sparsely populated mitochondria and randomly organized myofilaments^4^. Together with our data, we suggest that cardiac pacemaker cells are designed for sustaining viability at the cost of reduced ATP generation (Figs. 1 and 2), which is complemented by their low metabolic demand (Fig. 3). This idea makes teleological sense; cardiac pacemaker cells exhibit lower energy utilization compared to working myocardium, which promotes the principal function of these cells in generating spontaneous and oscillatory membrane depolarizations under metabolic challenges.

The maximal OCRs were substantially lower in TBX18-iPMs compared with the control on D3 and D5 but normalized back to the control level on D7 (Fig. 3a). The transient differences in the OCRs may be related to the process of somatic cell reprogramming by TBX18, a notion that deserves further investigation. Surprisingly, we found that small mitochondria and less-stringent metabolic demand are positively correlated with the synchronous automaticity of iPMs (Figs. 4 and 5). Our data indicate that the knockdown of Opa1, a mitochondrial inner membrane fusion factor for mitochondrial quality control^23^, enhanced the iPMs to pace together. This synchronization may owing to at least two mechanisms: hypoxic stress and the Opa1-mediated fragmentation of mitochondria could enhance the efficiency of somatic cell reprogramming by TBX18 and, consequently, the automaticity of the induced pacemaker cells. This potential mechanism is supported by an earlier study that has showed low O_2_ tension promoted the cellular reprogramming of fibroblasts into induced pluripotent stem cells^56,57^. Alternatively, the mitochondria could be in direct contact with the endo/sarcoplasmic reticulum (ER/SR) membrane, similar to the physical contact between the membranes of the two organelles^58–61^. Mitochondria-ER/SR contact sites have been shown to regulate the activity of ryanodine receptors (RyRs) and sarco-endoplasmic reticulum Ca^2+^-ATPases (SERCAs)^62–66^. Thus, the Ca^2+^ clock mechanism of automaticity might be enhanced in Opa1 knockdown TBX18-iPMs via direct contact with the mitochondria. These notions warrant further studies on the electrophysiological roles of factors that control mitochondrial membrane dynamics, such as Mfn1 and Mfn2, as well as Opa1.
